# Ancient DNA challenges prevailing interpretations of the Pompeii plaster casts

**DOI:** 10.1016/j.cub.2024.10.007

**Published:** 2024-11-07

**Authors:** Elena Pilli, Stefania Vai, Victoria C. Moses, Stefania Morelli, Martina Lari, Alessandra Modi, Maria Angela Diroma, Valeria Amoretti, Gabriel Zuchtriegel, Massimo Osanna, Douglas J. Kennett, Richard J. George, John Krigbaum, Nadin Rohland, Swapan Mallick, David Caramelli, David Reich, Alissa Mittnik

**Affiliations:** 1 Dipartimento di Biologia, Università di Firenze, 50122 Florence, Italy; 2 Department of History, Harvard University, Cambridge, MA 02138, USA; 3 Department of Human Evolutionary Biology, Harvard University, Cambridge, MA 02138, USA; 4 Dipartimento di Biologia, Università di Firenze, 50019 Florence, Italy; 5 Parco Archeologico di Pompei, 80045 Naples, Italy; 6 Ministry of Cultural Heritage and Activities and Tourism, 00197 Rome, Italy; 7 Department of Anthropology, University of California, Santa Barbara, Santa Barbara, CA 93106, USA; 8 Department of Anthropology, University of Florida, Gainesville, FL 32611, USA; 9 Department of Genetics, Harvard Medical School, Boston, MA 02115, USA; 10 Howard Hughes Medical Institute (HHMI), Harvard Medical School, Boston, MA 02115, USA; 11 Broad Institute of MIT and Harvard, Cambridge, MA 02142, USA; 12 Max Planck—Harvard Research Center for the Archaeoscience of the Ancient Mediterranean, 04103 Leipzig, Germany; 13 Max Planck—Harvard Research Center for the Archaeoscience of the Ancient Mediterranean, Cambridge, MA 02138, USA; 14 Department of Archaeogenetics, Max Planck Institute for Evolutionary Anthropology, 04103 Leipzig, Germany; 15 These authors contributed equally; 16 Lead contact

**Keywords:** ancient DNA, bioarchaeology, Roman Empire, strontium

## Abstract

The eruption of Somma-Vesuvius in 79 CE buried several nearby Roman towns, killing the inhabitants and burying under pumice lapilli and ash deposits a unique set of civil and private buildings, monuments, sculptures, paintings, and mosaics that provide a rich picture of life in the empire. The eruption also preserved the forms of many of the dying as the ash compacted around their bodies. While the soft tissue decayed, the outlines of the bodies remained and were recovered by excavators centuries later by filling the cavities with plaster. From skeletal material embedded in the casts, we generated genome-wide ancient DNA and strontium isotopic data to characterize the genetic relationships, sex, ancestry and mobility of five individuals. We show that the individuals’ sexes and family relationships do not match traditional interpretations, exemplifying how modern assumptions about gendered behaviors may not be reliable lenses through which to view data from the past. For example, an adult wearing a golden bracelet with a child on their lap–often interpreted as mother and child–is genetically an adult male biologically unrelated to the child. Similarly, a pair of individuals who were thought to have died in an embrace–often interpreted as sisters–included at least one genetic male. All Pompeiians with genome-wide data consistently derive their ancestry largely from recent immigrants from the eastern Mediterranean, as has also been seen in contemporaneous ancient genomes from the city of Rome, underscoring the cosmopolitanism of the Roman Empire in this period.

## Introduction

Pompeii was a Roman town located in Campania, Italy, 14 miles southeast of Naples. It was destroyed by the 79 CE Plinian eruption of Somma-Vesuvius, also known as the “Pompeii eruption”. The city was buried under a pumice lapilli deposit, laid down during the early phase of the eruption, followed by ash deposits from pyroclastic currents during a later phase. The town remained largely forgotten until its rediscovery in the late 1700s. Its unique preservation and the insight it provides into daily life in the Roman Empire has led to Pompeii becoming one of the world’s best-known archaeological sites and being designated as a UNESCO World Heritage Site.

The earliest stable settlements in the Gulf of Naples in the Iron Age date to the 8th century BCE, when the Osci built houses near the estuary of the river Sarno on a small hill, representing the remnants of multiple local volcanic vents, rising on the surrounding Sarno plain^1^. Due to its strategic location, Pompeii became an important road and port node. The Greeks, Etruscans, and Samnites all attempted to conquer Pompeii, and it eventually became a Roman colony.

The 79 CE eruption completely destroyed Pompeii, but the pyroclastic deposits that blanketed the city preserved its buildings, streets, and artifacts. Many bodies were also preserved, as were the art, jewelry, book rolls, and other cultural remnants of the inhabitants. During the excavations which began in 1748, numerous victims, both isolated and grouped, were found in the houses and in squares, gardens, and streets just outside the city walls^2–5^. In the 19th century, archaeologist Giuseppe Fiorelli developed a method of making casts by pouring liquid plaster into the voids left by decay of the victims’ soft tissue. Since then, over 1000 human victims have been discovered among the city’s ruins, and 104 plaster casts preserving the victims’ shapes and encasing their bones have been produced using Fiorelli’s method. In 2015, during restoration of 86 casts, an effort to CT scan or X-ray 26 of them revealed that none contained complete skeletons. The casts had been considerably manipulated and likely creatively restored in the past, with stylistic variations between casts in part reflecting aesthetic preferences of the periods in which they were made^6^. Popular interpretations of the identity of the victims in Pompeii therefore are influenced not only by the archaeologists first describing them, but also by the restorers who chose to enhance or alter some features of the bodies’ shapes. The imaging results revealed in some cases the introduction of stabilizing elements like metal rods, as well as the frequent removal of bones prior to the casting process, complicating the sex determination on an osteological basis. It was possible to reinterpret some casts such as the formerly imagined distended abdomen of a putative pregnant woman likely being formed by bunched up garments.

Multiple studies have confirmed the possibility of retrieving DNA data from both human and animal remains in Pompeii^7–17^. Recently, genetic data from human skeletal remains found in the *Casa del Fabbro*^18^ showed that this individual’s genetic ancestry fell within the genetic diversity observed in the Imperial Roman Latium (modern Lazio) region which had more Eastern Mediterranean influence compared to the preceding Iron Age^19^. As a port, Pompeii is typically viewed as a city with a diverse and mobile population. Bioarcheological analysis, however, revealed high frequencies of non-metric traits that may indicate genetic homogeneity or common environmental influences^5^. Ancient DNA and strontium isotopes offer the possibility of obtaining a better understanding of the diversity and origins of Pompeii’s residents.

We attempted to extract genetic information from the human plaster casts, using enrichment of ancient DNA extracts for mitochondrial DNA and more than a million single nucleotide polymorphism (SNP) targets^20^. The study was carried out on highly fragmented skeletal remains mixed with plaster recovered from different anatomical elements of 14 of the 86 casts undergoing restoration. Figure S1 presents a map showing where the 14 victims were found, and the casts made. Our goal was to test interpretations suggested in the absence of genetic data about the identity of the victims and their relations to each other, based on the shape and position of the bodies, and to enhance the information on osteological data previously obtained by X-ray and CT imaging of the mostly incomplete skeletons in the casts^6^. Such inferences have shaped how historians, archaeologists and the public imagine the society recorded so vividly by the catastrophe. In addition, we investigated the victims’ genetic ancestry and compared it with the known genetic diversity from contemporaneous individuals from the city of Rome and its hinterland.

## Results and Discussion

### DNA preservation and uniparentally inherited markers

In the following, we describe the individuals using the Cast Numbers^21^ which are commonly used in the bioarchaeological literature about the site. The corresponding Genetic IDs can be found in Table 1 and DataS1 A. We took samples of bone fragments mixed with plaster from 14 different casts (Table S1 and Figure S1) and screened them by quantifying the concentration and degradation of the DNA (Table S2), as well as using a hybridization-based approach to enrich for the mitochondrial DNA^22^ and 3,000 autosomal SNPs^23^ (Tables S3 and S4). On the basis of the screening results, we chose to enrich seven partially UDG-treated libraries for around 1.2 million nuclear SNPs (‘1240K’ SNP set) (DataS1 A). Additionally, we radiocarbon-dated four of the selected individuals (DataS1 B).

**Figure 1.**
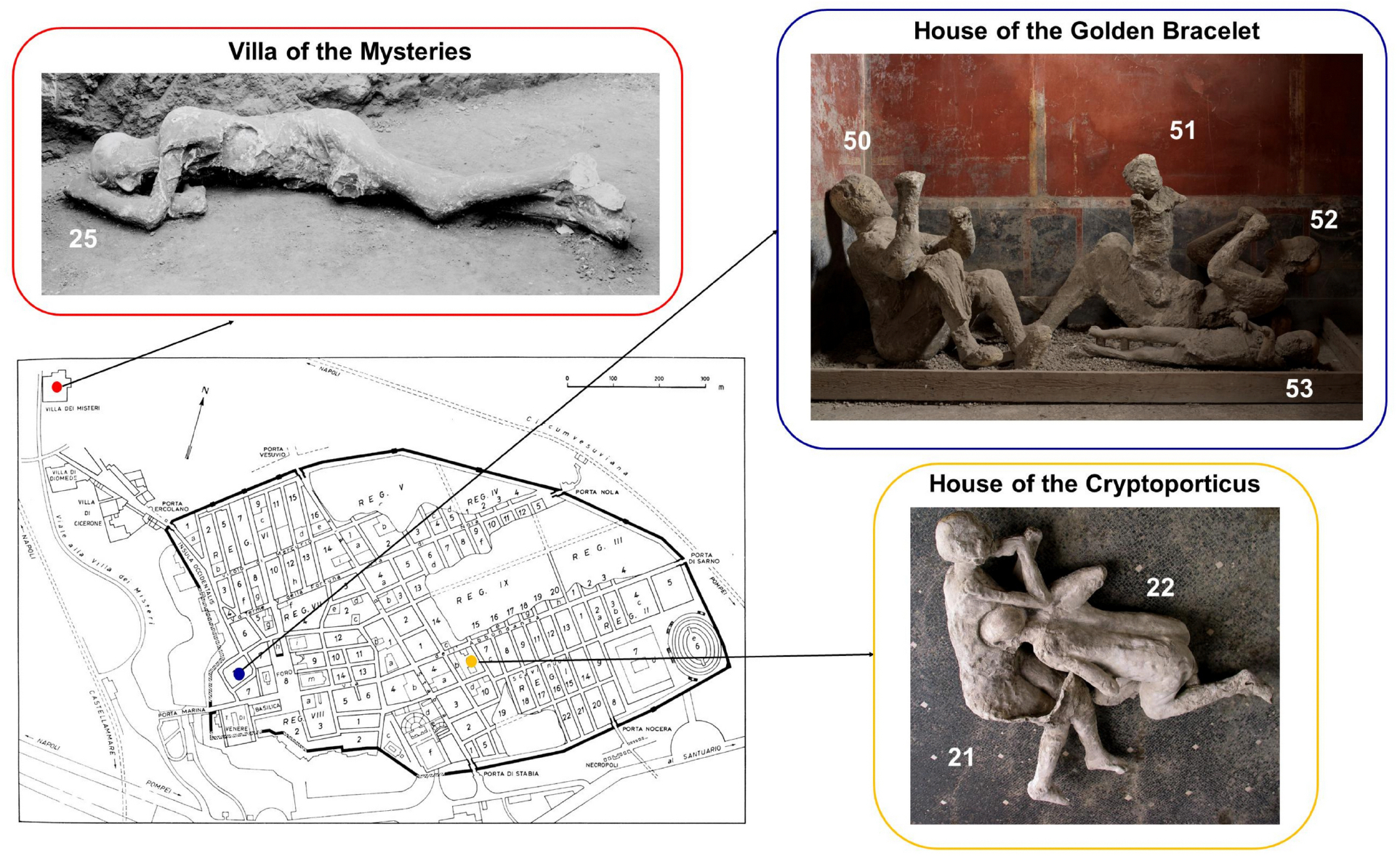
Pompeii plaster casts and their original locations in Pompeii. Plaster casts of individuals from whom analyzable ancient DNA was recovered and original map of Pompeii. See also Figure S1 and Table S1.

Five samples provided complete or partial mitochondrial genomes with patterns of base misincorporations at the read ends typical of ancient DNA (Table S3), and were covered on at least 50,000 of the targeted autosomal SNPs, with median coverage on the 1240K SNPs ranging from 0.006 X to 0.437 X (Table 1, DataS1 A). All five individuals (Figure 1) were genetically sexed as male as assessed by DNA quantification using the Quantifiler^™^ Trio Kit (STAR Methods). We also estimated contamination on the X chromosome for the two individuals for which there was sufficient data to make this quantification, and found it was below 4%. In the other cases the molecular damage pattern and contamination estimates provided by mitochondrial data (Table 1, Table S3, DataS1 A) indicate that the results are compatible with authentic ancient DNA originating from a single individual. All individuals were determined to belong to Y-chromosomal lineages (J2a, J2b, E1b and T1a) that first emerged in Western Asia and are today still found in the highest frequencies in Western and Central Asia, Southern Europe and North Africa^20,24–27^ (DataS1 C). None of the individuals had evidence of relatedness up to the third degree (DataS1 D).

### The plaster cast individuals represent an ancestrally diverse population

**Figure 2.**
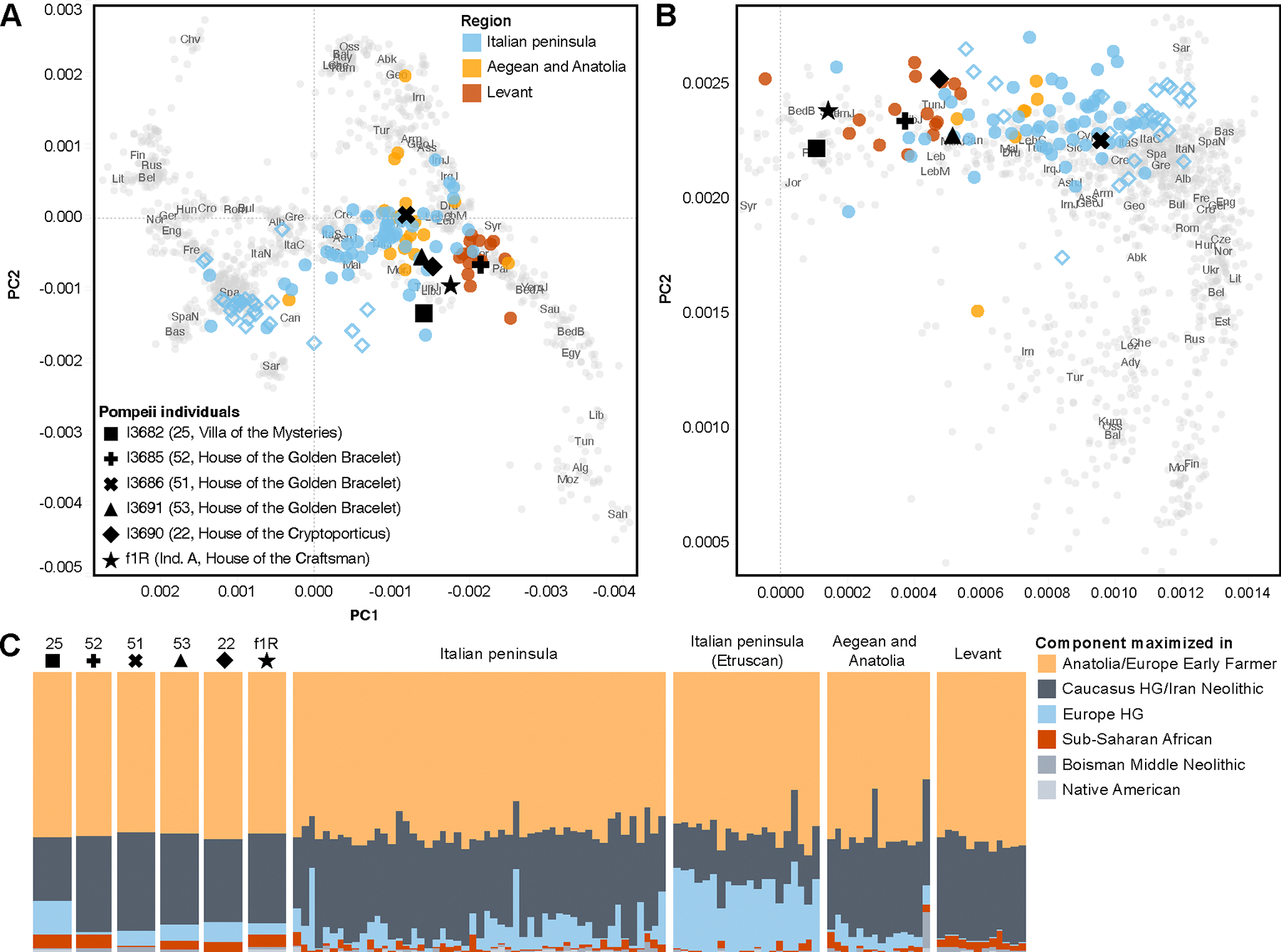
Principal components analysis and ADMIXTURE analysis. PC1 and PC2 were constructed on Human Origins genotyping data of modern-day West Eurasian and North African individuals **(A)** and a world-wide set of modern-day individuals **(B)**. Ancient individuals covered on at least 20,000 SNPs genotyped on the Affymetrix Human Origins array were projected onto them. Gray dots represent modern-day individuals, colored circles represent published individuals dated to between the 4^th^ century BCE and the 2^nd^ century CE, unfilled diamonds represent contemporaneous individuals from Etruscan cultural context in Northern and Central Italy, black symbols represent individuals from the context of the 79 CE Vesuvius eruption. **(C)** Results of unsupervised ADMIXTURE analysis, showing k=6 for Pompeian individuals and published ancient individuals (HG=Hunter-Gatherer). Full results for k=2 to k=15 shown in Figures S2 and S3.

We find that the five individuals are shifted away from modern-day Italians as well as Italian populations from the Iron Age and Late Iron Age and Imperial Period Etruscans on a Principal Component Analysis (PCA) constructed on modern-day West Eurasian and North African populations and world-wide populations. Instead, they cluster more with eastern Mediterranean, Levantine and North African Jewish populations (Figures 2A and B). This pattern is similar to the one found for the Imperial Roman population of Central Italy^19^ and the previously published single individual from Pompei with genome-wide data, which we co-plotted with the five individuals with newly generated data^18^. In an unsupervised ADMIXTURE analysis at k=6 (Figure 2C, Figures S2 and S3), the Pompeian individuals differ from the roughly contemporaneous individuals associated with the Etruscan culture in their proportions of the ancestry components maximized in Mesolithic European hunter-gatherers (lower in the Pompeiians) as well as hunter-gatherers from the Caucasus (higher in the Pompeiians), making their ancestry composition more similar to the Central Italian Imperial Romans as well as contemporaneous individuals from the Aegean and Anatolia. Individual 52 shows an ancestry composition comparable to Iron Age and Roman period Levantine individuals, characterized by a minor component maximized in modern-day Sub-Saharan African populations and an absence of the component maximized in European hunter-gatherers. This individual most closely clusters with Levantine populations.

**Figure 3.**
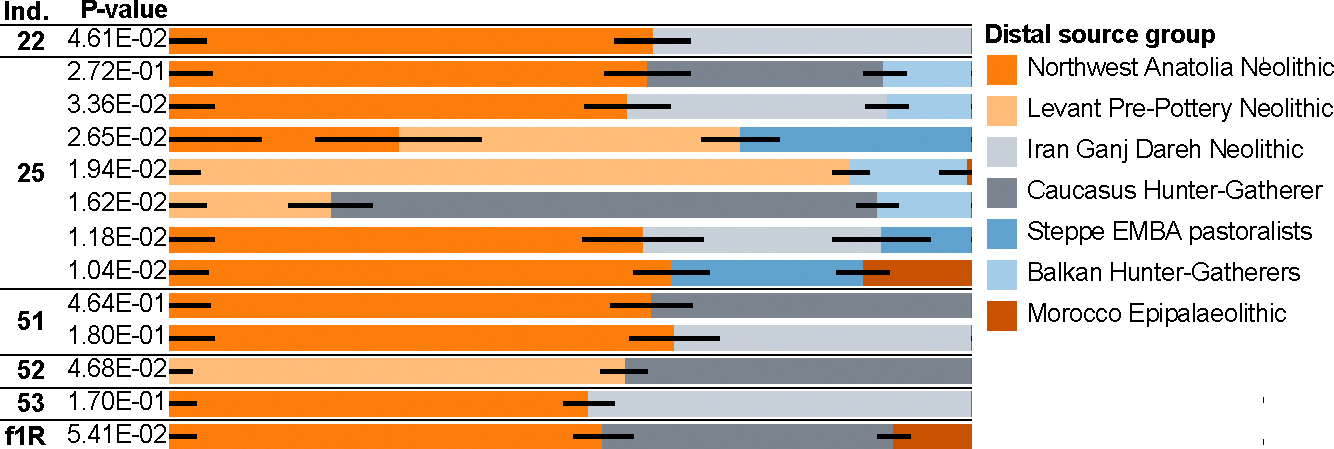
Distal *qpAdm* models. Working qpAdm models using distal ancestry sources with p>0.01. See also DataS2 A and B.

To quantify the contribution of different ancestry sources, we tested various 2- and 3-way models using *qpAdm* and report the working models using the least number of sources with *P*>0.01 and coefficients below 1 (DataS2 A and B). Using a distal set of putative ancestral populations, we found working models for all individuals (Figure 3). For each individual from the casts, ancestry related to Anatolia Neolithic farmers (TUR_Marmara_Barcin_N) and/or Levantine Pre-Pottery Neolithic farmers (Levant_PPN) compose the largest inferred proportion (48–75%), while the second largest proportion is inferred to derive from people related to Neolithic farmers from Iran (IRN_Ganj-Dareh_N) (26–45%), with exception of individual 25 who can be modeled as deriving 65.3 ± 4.5% of his ancestry from Levantine Pre-Pottery Neolithic farmers (Levant_PPN) and 34.7 ± 4.5% from Bronze Age steppe pastoralists (Steppe_EMBA), an ancestry source required by none of the other individuals. This model indicates, despite the low coverage of the individual’s genome, a different ancestry history involving European sources that did not contribute to the other individuals.

Individuals 22 and 51 can be described as comprised of around 62–69% Anatolia Neolithic farmers (TUR_Marmara_Barcin_N) and 31–38% Neolithic farmers from Iran (IRN_Ganj-Dareh_N) ancestry. Individual 52 is inferred to have no Anatolia Neolithic farmer (TUR_Marmara_Barcin_N) ancestry. Instead, he can be modeled as deriving 57.7 ± 3.1% and 42.3 ± 3.1% of his ancestry from Levantine Pre-Pottery Neolithic farmers (Levant_PPN) and Neolithic farmers from Iran (IRN_Ganj-Dareh_N), respectively. This result corroborates the closer clustering of this individual with contemporaneous Levantine individuals in PCA and ADMIXTURE analysis (Figure 3) and suggests a recent Levantine origin for him or his direct ancestors. Two alternative 3-way models are not rejected for individual 53. In both the largest proportion of ancestry is accounted for by the combination of components derived from Neolithic farmers from Iran (IRN_Ganj-Dareh_N) and Anatolia Neolithic farmers (TUR_Marmara_Barcin_N) – 69–88% and 84–97%, respectively. The third component is derived either from Neolithic farmers from the Levant (Levant_PPN) with 21.2 ± 7.1%, or Chalcolithic individuals with North African ancestry (Mediterranean_CA_NorthAfrican), with 9.8 ± 2.8%.

We also tested the individuals as potentially derived from more proximal sources consisting of late 2^nd^ BCE to early 1^st^ millennium CE individuals from different West Eurasian and North African regions that, within their grouping, are relatively genetically homogeneous according to the PCA (DataS2 C and D). The working models (*P*>0.01), largely require Eastern Mediterranean, Levantine or North African populations as the main source (DataS2 C). Models with ancestry indistinguishable from one of the sources Turkey_West and Turkey Central work for Individual 22 and Individual 51. Working 2-way models for individual 25 involve an eastern Mediterranean source which contributed the majority of ancestry in combination with a Balkan, Central Mediterranean or Central European source. Similarly, Individual 53 can only be fit as a mixture of ancestry sources, with three tested models, ~80% Turkey_Central with 20% Sardinia_Punic, ~67% Turkey_East with ~33% Sardinia_Punic and ~80% Lebanon with ~20% Italy_IA (Iron Age individuals from the Italian peninsula) all fitting the data. No working 1- or 2-way model was found for individual 52, but the feasible models with the highest *P*-values (between 1.64E-3 and 2.321E-6) all contain Egypt as the source contributing the majority of the ancestry (~51–73%), suggesting the actual source is genetically similar to the one represented by the Hellenistic Egyptian individuals (DataS2 D). The previously published Individual f1R requires for all working models about 73–84% from the Lebanon source, in combination with a European source.

### Demographic and phenotypic characteristics

Only individual 52 provided enough genetic data to detect runs-of-homozygosity (ROH), which can give insight into effective populations size and marriage practices^28^. Only a single short ROH of 4.71 cM was found in this individual (Figure S4), inconsistent with closely related parents or a small population of origin in which individuals share marked background relatedness. Overall, the genetic heterogeneity seen in the sample of genomes from Pompeii and the absence of long ROH in one individual are consistent with a large, diverse population, as is suggested by its role as a river port and its description as being composed of various local and immigrant cultural groups by ancient authors^5^. This provides some support for the hypothesis that the high frequencies of some non-metric traits observed among the 79 CE eruption victims could be due to shared environmental variables during developmental stages, rather than a common genetic background. However, among the individuals we studied genetically, only individual 22 exhibited such common non-metric traits, specifically the presence of relatively large ossicles at lambda and Wormian bones in the lambdoid suture^5^. Targeted genetic analyses of individuals with and without such traits might reveal a level of homogeneity in ancestry among the former.

Phenotypic markers for externally visible characteristics, assessed with the HIrisPlex-S system^29^, suggest highest probabilities for brown eyes for individuals 25, 51 and 53, and dark skin and black hair for individual 52 (DataS3 A). Several risk alleles associated with different diseases were detected in all the samples analysed but the low coverage and possible interaction between genotype and environment did not allow us to predict any of these traits with certainty (DataS3 B–G).

### Combined genetic and osteological findings contradict some of the established popular narratives

Scholars as well as the public’s imagination have interpreted the impressions of body position and shape in the plaster casts in diverse ways, speculating about the identities of the victims and their potential connections to each other. When the restoration and the study of the casts through new scientific approaches began in 2015, there were no peer-reviewed osteological references providing sound data for an individual characterization and the only information available was that provided by the archaeologists. Nevertheless, some interpretations about sex and relationships between individuals found wide appeal with the scholars’ community and the public, being spread through museal exhibitions and educational and publicity materials. Therefore, integrating our newly reported genetic and isotopic data allows us to revisit some of the widespread interpretations.

#### House of the Golden Bracelet

The House of the Golden Bracelet is situated in Insula 17, Regio VI (Figure 1). The house’s innovative and complex architectural form resulted from a fusion between the Roman-Italic model of the atrium house and that of the suburban villa. Built on three levels, it utilized the city walls and the slope of the hillside. Its rooms were arranged in terraces on the three levels in a panoramic position. The house was especially rich, and colorful frescoes decorated its walls. In 1974, four victims were discovered in close vicinity to each other at the site and were interpreted as constituting a genetically related family^30^. The first three victims were found at the foot of the staircase that led to the garden and seafront: two adults (Individuals 50 and 52) and a young child (Individual 51), apparently standing on Individual 52’s hip. Individual 52 was traditionally interpreted as a woman and mother, due to the association with the child, and an intricate golden bracelet of exceptional weight (6.1 grams) worn on one arm, which also gave the house its name. The other adult, Individual 50, was interpreted as the father in the genetically related family group. On the basis of limited X-ray analysis, Individuals 50, 51 and 52 were estimated to be a young adult, a 5 to 6-year-old and a younger middle-aged adult, respectively, but a clear sex attribution could not be made for either of the adults or the child^6^. It has been suggested that these three victims sought refuge in the stairwell and were killed by the staircase that collapsed as they tried to flee their residence for the city’s port^30^. A few meters away, a body of a child about 4 years old was found (Individual 53), interpreted as a boy due to a bulge in the plaster in the area of the genitalia^30^. This child was plausibly separated from the family group during the escape along the corridor leading to the garden.

DNA quantification allowed us to estimate the molecular sex of all the individuals, while sex identification on the basis of nuclear genome analysis was only possible for three of the individuals (all but individual 50). All individuals were sexed as genetically male, including the presumed female adult (Individual 52). Furthermore, both the mitochondrial DNA and whole genome data found no evidence of biological relatedness, at least up to the third degree, between any of the individuals, falsifying the prevailing narrative of these four victims as a genetically related family. The preservation of genetic material in the adult individual 50 allowed us to reconstruct only the mitochondrial genome, showing no matrilineal relations with the other three individuals, although we cannot exclude that he could be related to them at the nuclear level. The PCA and ADMIXTURE analyses show a considerable variation in the distribution of these three individuals within the genetic diversity observed for the Italian inhabitants of Imperial Rome (Figure 2)^19^. The composition of genetic ancestry of the three individuals inferred by *qpAdm* appears distinct in both distal and proximal modeling (DataS2), which suggests their respective ancestors had origins in different Eastern Mediterranean or North African populations. Trying to reconstruct the appearance of these individuals by inferring phenotypes based on genotypes, we found that individual 52 had black hair and dark skin, while we were able to attest only the eye color for individuals 51 and 53, which was brown (DataS3 A).

#### House of the Cryptoporticus

The House of the Cryptoporticus is situated in Insula 6, Regio I (Figure 1). The house was originally built in the 3^rd^ century BCE. It takes its current name from the *cryptoporticus*, an underground passageway with openings, running along three sides of the quadrangular south-opening garden. A living room (the *oecus*) and four thermal bathing rooms (*apodyterium, frigidarium, tepidarium* and *calidarium*, the latter being preceded by a *praefurnium*) open onto it. The *cryptoporticus* originally had barrel and cross vaulted ceilings and the walls of the *oecus* were decorated with a series of scenes inspired by the Iliad, providing one of the finest examples of Pompeian painting from the final stage of the Second style (era of Augustus). The walls of the four bathing rooms were also painted with exquisite scenic images. During the excavations in 1914, nine individuals were found in the garden in front of the house, but it was only possible to produce casts for four of them. Among these four individuals, two (Individuals 21 and 22) were found close to each other in what was interpreted as an embrace (Figure 1) and the archaeologists hypothesized that they could be two sisters, mother and daughter, or lovers^21,31–34^. CT scanning of skeletal elements preserved within the casts led to an age estimate of 14–19 for Individual 21 and a young adult age for Individual 22. Osteological sex estimation was not possible but the relative gracility of Individual 22’s skull was noted^6^. The nuclear genetic analysis was successful only for Individual 22 and revealed he was male, excluding the possibility that the pair of victims were sisters or mother and daughter. Like all the other analyzed samples, the individual falls within Mediterranean nuclear genetic variability, with an ancestral origin consistent with contemporaneous Anatolian populations (DataS2 C), and carries the mitochondrial haplogroup N1b1a1, with a presumably Near Eastern/North African origin^35,36^. Reconstruction of the mitochondrial genome was successful also for individual 21 (DataS1), which carries the two derived SNPs of the distinct haplogroup R and none of the derived SNPs leading to N1b1a1, which is consistent with a lack of maternal relatedness between the two individuals.

#### Villa of the Mysteries

**Figure 4.**
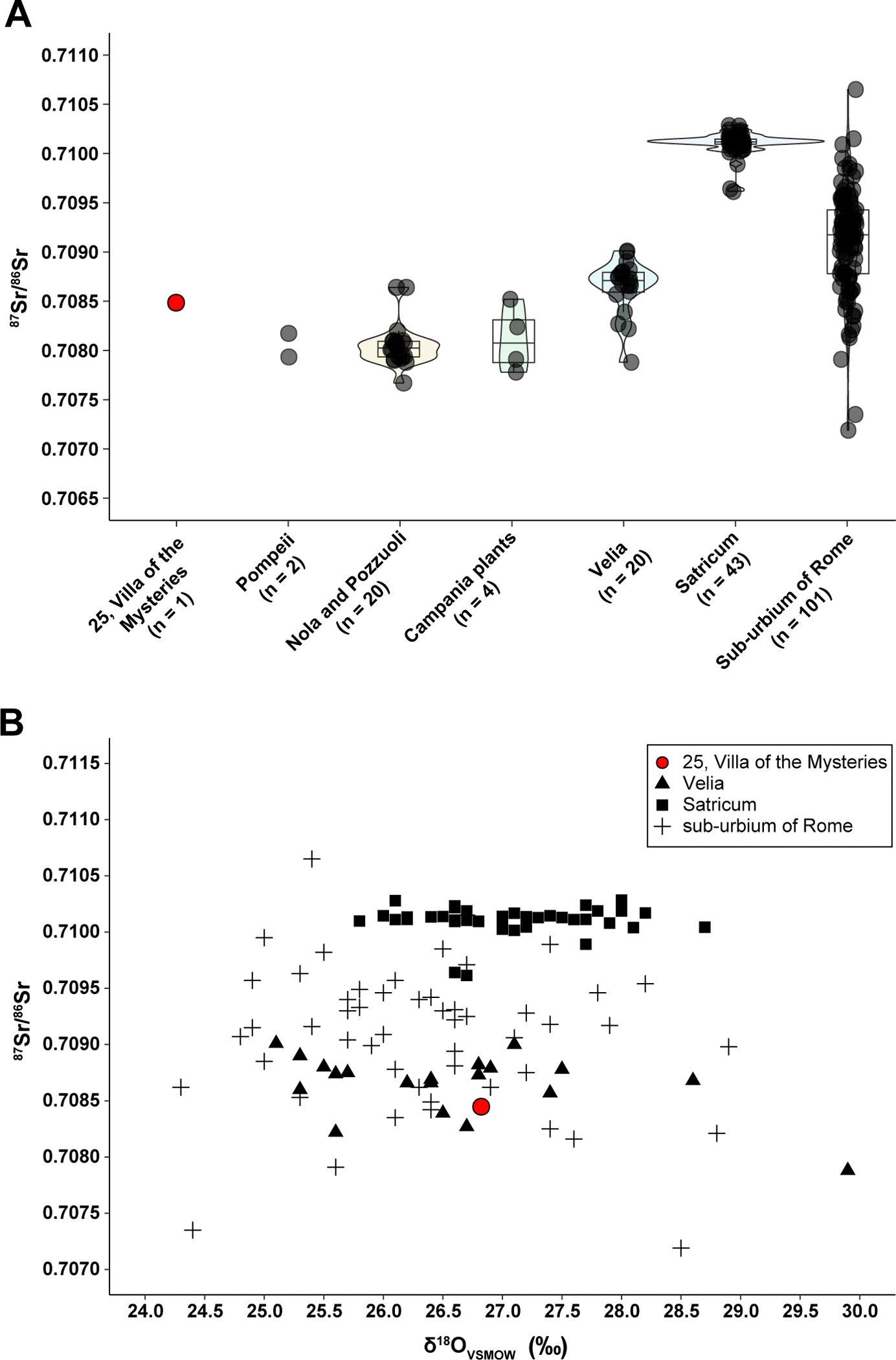
Strontium and oxygen analysis of Individual 25 from *Villa of the Mysteries*. **(A)** Bioavailable strontium distributions comparing the Individual 25 (this study, red dot), Pompeii^38,39^ Nola & Pozzuoli with plant samples from the southern Campania plains^39^, the sub-urbium of Rome^40^, Satricum located southeast of Rome^41^, and Velia located on the western coast of southern Italy^44^. Violin and box plots show the similarities between sites located in central Italy and differences between populations near Rome. The *Villa dei Misteri* results fall within Sr ranges found across the Campania region and more broadly within ranges across the central and southern Italian peninsula. **(B)** Bioavailable strontium and oxygen isotope data for Individual 25 compared with published isotope data (n = 118) from the sub-urbium of Rome^40^, Satricum located southeast of Rome^41^, and Velia located on the western coast of southern Italy^44^. The Villa dei Misteri individual shares isotopic affinities consistent with Sr and O ranges found across the central Italian peninsula (see^38,42^). No published oxygen isotope data is currently available from Pompeii and the surrounding sites. Sr values higher than 0.7115 and lower than 0.7065 are not displayed in figure (see^40^). See also STAR Methods.

This villa, which was first excavated in 1909–1910 and is still subject to minor investigations arising from protection and conservation concerns today, is located northwest of the town walls, near the ancient seashore (Figure 1). Most of its walls, ceilings, and particularly its frescoes survived the eruption of the Vesuvius largely intact. The name (*Villa dei Misteri*) comes from a series of frescoes dating back to the 1^st^ century BCE, depicting a ritual probably dedicated to Bacchus, the god of wine, fertility, and religious ecstasy. The villa was very large with many different rooms and functional spaces, as was common for many Roman villas of that period. A wine press was found and restored in its original location, reflecting the fact that it was common for wealthy families to produce their own wine, olive oil, and other products since most villas included some farmland. The bodies of two adults, interpreted as women, and a child were found in the pumice lapilli deposit indicating they were caught in the early stages of the eruption on the upper floor of the farm section and fell to the lower floor. Six bodies were found in the overlaying ash deposits indicating they had survived the first phase of the eruption. Among them, Individual 25 was found alone in a room, lying atop a layer of ash, with an iron ring with an engraved carnelian of a female figurine on the little finger of the left hand, five bronze coins and a whip as personal effects^37^ (Figure S1). The cast of this victim shows some of the most well preserved anatomical and textile details. The man was about 1,85 m tall, thin, with a convex nasal bridge. According to the traces of his clothes and the ornaments, he was supposed to belong to a low social status and was interpreted as the custodian of the villa who had faithfully remained at his post^21^. Our genetic analysis confirms a male sex estimation, and mixed genetic ancestry which could possibly be traced to both Eastern Mediterranean and European sources (DataS2 C). To learn more about this individual’s geographic origin and lifetime mobility, we conducted strontium and oxygen isotope analysis (Figure 4). Although the strontium measurement (^87^Sr/^86^Sr = 0.7084729 +/− 0.00001) is higher than values found at Pompeii (μ = 0.70806, n=2)^38^, this value is consistent with the bioavailable Sr range found across the southern Campania plain (Figure 4A (0.7075–0.7085)^39^). This is outside of the local range described in the Roman population of Latio^40,41^ and regions across Northern and Southern Italy^38,42,43^. The δ^18^O_enamel_ composition (δ^18^O _VSMOW_ = 26.77 ‰, δ^18^O _VPDB_ = −4.03 ‰) is consistent with coastal distributions of δ^18^O values in the central Italian peninsula (Figure 4B)^38,42,44^. Isotopic affinities with bioavailable ^87^Sr/^86^Sr and δ^18^O across the central Italian peninsula potentially indicate early residency in and around Pompeii; however, while this assessment suggests local origins that fall within the expected local ranges, similarities in geologic and bioavailable isotope systems are found across the Mediterranean^45,46^.

Besides emphasizing the cosmopolitanism and mobility that shaped urban Roman Imperial populations, this study illustrates how unreliable narratives based on limited evidence can be, often reflecting the worldview of the researchers at the time. In this light, genetic analysis can greatly enrich these narratives when integrated with archaeological data. For example, at two of the villas we analyzed, individuals previously assumed to be women, in absence of careful osteological assessment, were found to be genetically male. These discoveries challenge longstanding interpretations, such as associating jewelry with femininity or interpreting physical closeness as indicators of biological relationships. Similarly, the genetic data complicate simple narratives of kinship: at the House of the Golden Bracelet which is the only site for which we have genetic data from multiple individuals, the four individuals commonly interpreted as parents and their two children, are in fact not genetically related. Instead of establishing new narratives that might also misrepresent these people’s lived experiences, these results encourage reflection on conceptions and construction of gender and family in past societies as well as in academic discourse. Furthermore, it is possible that the exploitation of the casts as vehicles for storytelling led to the manipulation of their poses and relative positioning by restorers in the past. Genetic data, together with other bioarchaeological approaches, provide the opportunity to deepen our understanding of the people who became victim of the Vesuvius eruption and highlight how integrating genetic data with archaeological and historical information, even in a historically rich site like Pompeii, significantly enhances our understanding of past lives and behaviors.

## STAR Methods

### Resource availability

#### Lead contact

- Further information and requests for resources and reagents should be directed to and will be fulfilled by the lead contact, Alissa Mittnik (alissa_mittnik@eva.mpg.de).

#### Materials availability

- This study did not generate new unique reagents.

#### Data and code availability

- All data needed to evaluate the conclusions in the paper are present in the paper and/or the supplemental information.
- Newly reported ancient sequencing data have been deposited at European Nucleotide Archive (ENA) and are publicly available as of the date of publication with the following accession number ENA:PRJEB74999. Haploid genotypes for the 1240k panel for the newly reported ancient individuals, and genotype data for the newly reported present-day individuals are available at https://reich.hms.harvard.edu/datasets.
- This paper does not report original code.
- Any additional information required to reanalyze the data reported in this work paper is available from the lead contact upon request.

### Experimental model and study participant details

#### Ancient individuals

- A description of the archaeological context of the ancient human individuals analyzed is provided in STAR Methods, Table S1 and Figure S1.

### Method details

#### Plaster casts

In 79 CE the Mount Somma eruption that led to the formation of Vesuvius, destroyed Pompeii and killed the entire population. As the people slowly died from the painful hot and lethal gases and/or ash, they were covered with pumice and ash. Subsequently, the rainfall caused the bodies to become cemented in the ash and while the soft tissues decomposed, the hardened ash preserved the outline of the bodies. The impressive excavations to unearth the Pompeii city began in 1748 and proceeded sporadically, until the archaeologist Giuseppe Fiorelli began to carry out the research systematically, keeping detailed records of all the findings. In 1863 Fiorelli set up a method to realize plaster casts on some of the victims of the eruption, pouring liquid chalk into the voids left by the bodies in the hardened ash^47^. This method allowed to recreate the shape of the bodies even with their expression at the time of death (Figure S1). Plaster casts were made for 104 of the estimated approximately 1000 victims found in Pompeii.

#### Sampling of skeletal material and DNA extraction

During the recent efforts of cast restoration at the Pompeii Archaeological Park, we had the possibility to collect bone fragments and teeth from inside the casts. The skeletal samples were accessible through damaged parts of the casts, and they showed different degrees of preservation. In some cases, the bone material was mixed with plaster and highly fragmented and fragile (Figure S1). A first set of samples (Set 1: Cast Numbers 21, 22, 50, 51, 52 and 53) from six individuals from the House of Cryptoporticus and the House of the Golden Bracelet were chosen for molecular analysis to verify whether the reconstruction of the relations between them made on an archaeological basis was supported. Later on a further set of samples from eight additional individuals (Set 2) was collected (Table S1).

Sampling, DNA extraction and library preparation of all specimens was carried out in the Molecular Anthropology Unit of the University of Florence, a state-of-the-art facility dedicated to the analysis of ancient DNA samples. To remove potential contamination, the outer layer of the bone fragments and teeth was mechanically removed using a rotary sanding tool (Dremel^®^ 300 series). After brushing, each sample was irradiated by ultraviolet light (λ = 254 nm) for 45min in a Biolink DNA Crosslinker (Biometra). DNA was extracted from approximately 50 mg of powder collected from the bones or from the tooth root following a protocol designed for optimizing the retrieval of very short DNA fragments in highly degraded samples^48^.

#### Quantification and evaluation of DNA preservation

DNA extracts of Set 1 were quantified in duplicate at the University of Florence, to obtain a preliminary description of the molecular preservation of such peculiar material. The presence of inhibitors and the DNA degradation level were evaluated using the Quantifiler^™^ Trio DNA Quantification Kit (Thermo Fisher Scientific, Oyster Point, CA). Real-time PCR amplification reactions contained 10.5 μL of Primer-Probe Mix, 12.5 μL of Master Mix, and 2.0 μL of the DNA sample as per the user’s manual^49^. The data were analyzed using the HID Real-Time PCR Analysis Software v1.2 with the settings provided. The quantification results are summarized in Table S2. No results were obtained from sample 21 for all targets analyzed. The quantification values obtained from samples 22 and 50 were zero for the large autosomal target, presumably due to DNA degradation in too-short fragments. Similar results were achieved for the Y marker in which the concentration varied between 2.8 and 107 pg/μl for all five samples. Positive results for the Y marker suggested that the samples were male. Because no results were obtained for the large autosomal marker from the two samples, the degradation index (DI) measuring typical ancient DNA contamination could only be computed reliably for samples 52, 53, and 51. The estimated DI values for these samples were 19.2, 7.6, and 14.8, respectively. As reported in the user manual^49^, these DI values indicate that the DNA in the samples was moderately/significantly degraded, consistent with authentic ancient DNA. No significant shift from the non-template control in IPC CT was observed, indicating that if inhibition was present, it was not enough to suppress IPC amplification significantly.

#### DNA library preparation

Illumina NGS libraries were constructed starting from 20 μl of DNA extract each, following a double-stranded DNA protocol using a unique combination of two indexes per specimen. Libraries without any UDG treatment were made for Set 1^50^, to allow for assessment of the deamination patterns as a criterium of authenticity. We produced libraries with partial-UDG treatment for all the 14 samples^51^ for screening and potential 1240K SNP capture. Negative controls were checked with both qPCR and Agilent 2100 Bioanalyzer DNA 1000 chip. After adapter ligation blanks had a concentration of 4–5 order lower than the biological samples, while indexing PCR products showed the presence of adapter-indexes dimers only.

#### Mitochondrial DNA capture and sequencing

At the University of Florence, the non-UDG-treated libraries of Set 1, along with extraction and library blanks, were enriched for mitochondrial DNA following a multiplexed capture protocol^22^ and sequenced on an Illumina MiSeq instrument for 2×76+8+8 cycles. After demultiplexing, raw sequence data were analysed using a pipeline specific for ancient DNA samples^52^, using the following tools implemented in the pipeline. Adapters were clipped-off and paired-end reads with a minimum overlap of 10 bp merged in a single sequence using Clip&Merge version 1.7.4. Merged reads were then mapped on the revised Cambridge Reference Sequence, rCRS (GenBank Accession Number NC_012920) by CircularMapper in order to take into account the circularity of the mitochondrial genome. Duplicates were removed using DeDup, a tool that considers both ends of the fragments to recognize them as clonal. Reads with mapping quality below 30 were discarded.

Mapping results of these samples are shown in Table S3. Samples 22, 50, 51, 52, and 53 presented a mean coverage of 55.39, 37.34, 3.43 40.82, and 69.85 respectively, with more than 99% of the mitochondrial genome covered at least by 5 sequences with the exception of sample 51 with lower coverage (more than 80% of the mitochondrial genome covered at least by 2 sequences). No usable data was obtained for sample 21.

#### Second mitochondrial DNA capture, autosomal capture and sequencing

We screened aliquots of all 14 prepared partial-UDG-treated libraries as well as three extraction and library blanks at the ancient DNA facilities of Harvard Medical School, Boston MA, USA, by in-solution hybridization, enriching for the mitochondrial genome^53^, along with about 3,000 nuclear SNPs using a previously described bead-based capture^23,54^ with probes replaced by amplified oligonucleotides synthesized by CustomArray Inc. After the capture, we completed the adapter sites using PCR, attaching dual index combinations to each enriched library. We sequenced the screening products on an Illumina NextSeq500 using v.2 150 cycle kits for 2 × 76 cycles and 2 × 7 cycles. Screening results are summarized in DataS1 A.

For the seven libraries that passed the screening performed at Harvard Medical School, we performed 1240K capture, using two rounds of in-solution enrichment on the targeted set of 1,237,207 SNPs using previously reported protocols^55,56^. After indexing the enrichment products in a way that assigned a unique index combination to each library^57^, we sequenced the enriched products on an Illumina NextSeq500 instrument using v.2 150 cycle kits for 2 × 76 cycles and 2 × 7 cycles.

#### Data processing

We trimmed barcodes and adapters from the raw sequences and merged read pairs with at least 10 or 15 overlapping base pairs according to the length of the sequenced reads and mapped the resulting reads to the human reference genome, hg19 [GRCh37] using the *samse* command of BWA (version 0.6.1)^58^.

Four samples provide complete or nearly complete mitochondrial genomes after the mtDNA target enrichment performed on non-UDG-treated libraries prepared in Florence on the first batch of six samples collected. We assessed the authenticity of these mitochondrial genomes by examining the characteristic aDNA damage patterns^59^ and confirming a single source for mitochondrial sequences using *contamMix*^60^ (Table S3). The consensus sequences of the individuals were uploaded to Haplogrep3^61^ to determine mtDNA haplogroups. Mitochondrial haplogroups were also determined for the data obtained from the partial UDG-treated libraries on all the 14 samples through Haplogrep3^61^. For the samples processed in both laboratories, the mitochondrial profiles obtained from the two different library protocols were compared and provided further support to the authenticity of the data.

We determined genetic sex on the 1240K capture data using sexDetERRmine^62^. Of the seven individuals that passed screening, we were able to determine that five were genetically male (consistent with one X and one Y chromosome and inconsistent with two XX chromosomes and no Y chromosome), while two individuals remained indeterminate due to low coverage (DataS1 A). Damage patterns as assessed with MapDamage 2.0^59^ were lower than in the data generated in the mtDNA capture performed in Florence, which is expected due to the partial UDG-treatment used on the 1240K capture libraries (Table 1). Sex estimates remained constant within the resolution of the confidence intervals after retaining only reads with C-to-T and G-to-A misincorporations at the 5’ and 3’ ends, respectively, using pmdtools v0.60^63^ (DataS1 A). We estimated heterozygosity on the X-chromosome of males using ANGSD^64^. For two males with sufficient coverage of at least 200 informative SNPs on the X chromosome we estimated nuclear contamination rates below 4%.

The individuals with working data had a median coverage on the 1240K SNP set of 0.054 (range 0.006 – 0.437). We prepared a genotype dataset for population genetic analysis by using mapped sequences with two bases trimmed from se ends by choosing one allele at random at the 1240K capture sites and retained five individuals for analysis that had at least 10,000 SNPs covered at least once (range 53,739 – 364,533).

We assigned Y-chromosomal haplogroups according to the Yfull 8.09 phylogeny using all trimmed reads mapped to the Y chromosome and report the most downstream diagnostic SNPs (DataS1 C).

#### Population genetic and relatedness analysis

We compiled a reference dataset consisting of whole genome data from 2,674 ancient individuals^65^ as well as previously reported whole-genome sequencing data from 346 worldwide modern-day individuals^66–69^ and merged the five newly reported pseudo-haploid genotypes from Pompeii.

We merged this dataset with 3291 modern-day individuals from 109 worldwide populations genotyped on the Affymetrix Human Origins (HO) SNP^54,70–74^. We used the *smartpca* function of EIGENSOFT (57) to perform principal component analysis (PCA) using default parameters, with the settings *lsqproject:YES* and *numoutlier:0*. We projected the ancient individuals onto a PCA plot of 1196 modern-day West Eurasian individuals, and 1900 modern-day worldwide individuals, restricting to the HO set of 597,573 SNPs.

We performed clustering using unsupervised ADMIXTURE^75^, after pruning SNPs in linkage disequilibrium with one another with PLINK^58^ using the parameter *--indep-pairwise 200 25 0.4*, which left us with 282,184 SNPs. We performed an ADMIXTURE analysis for values of k between 2 and 15, carrying out 5 replicates at each value of *k* and retaining the highest likelihood replicate at each *k* (Figures S2 and S3).

We performed *qpWave*^10,11^/*qpAdm*^10^ analyses with default parameters and *allsnps: YES*, precomputing *f*-statistics with using *qpfstats* (https://github.com/DReichLab/AdmixTools/blob/master/qpfs.pdf). All tools are implemented in ADMIXTOOLS. As “right” outgroups we used a set of 9 populations: “OldAfrica” (a diverse set of ancient African individuals with no evidence of West Eurasian-related admixture^76–78^), MAR_Taforalt_EpiP (Epipaleolithic North Africans^79^), RUS_AfontovaGora3 (Mesolithic hunter-gatherer from Siberia^80^), RUS_EHG (Hunter-gatherers from north-eastern Europe^55,81^), GEO_CHG.SG (hunter-gatherer from the Caucasus^82^), WHGB (Hunter-gatherers from the eastern Baltic and the Balkans^55,81^), ISR_Natufian_EpiP (Levantine Epipaleolithic hunter-gatherers^54^), TUR_EpiPal_Pinarbasi (an Anatolian Epipaleolithic hunter-gatherer^83^) and TUR_C_Boncuklu_PPN (Anatolian pre-ceramic farmers^83^). As distal source populations we used TUR_Marmara_Barcin_N (North-Western Anatolian Neolithic farmers^55^), Mediterranean_CA_NorthAfrican (two individuals from Chalcolithic Iberia and Sardinia with fully North African ancestry^84,85^), WHGA (hunter-gatherers from Western and Central Europe^20,55,80,86–88^), Levant_PPN (Levantine pre-ceramic farmers^54^), Steppe_EMBA (pastoralists from the Pontic-Caspian steppes associated with the Yamnaya and Poltavka cultural complexes^20,55,89^), and IRN_Ganj_Dareh_N (Neolithic farmers from the Zagros region^54,90^). In the modeling using proximal source populations^19,20,24,55,70,71,85,89,91–99^, we replaced GEO_CHG.SG with IRN_Ganj_Dareh_N in the outgroups so as to avoid a batch effect of attraction between sources and outgroups produced with the same processing strategy. We additionally added TUR_Marmara_Barcin_N and Steppe_EMBA to the outgroups and applied a “competitive” approach, adding unused sources to the outgroups. Corresponding genetic IDs for all outgroups and source groups are listed in DataS2 A.

We estimated relatedness between the Pompeian individuals using KIN^10075^ with default parameters, and BREAD R^101^ specifying a distance of at least 50,000 bp between overlapping sites. Results are shown in DataS1 D.

We used hapROH^28^ on the individual covered at more than 350,000 SNPs to detect Runs of Homozygosity (Figure S4).

#### Prediction of phenotypic traits

We explored the genomic data for the five previously selected individuals to predict phenotypic traits related to diseases, and externally visible characteristics (EVCs). We collected information about genetically determined medical conditions available in SNPedia (https://www.snpedia.com/index.php/Category:Is_a_medical_condition updated in October 2023) and sorted in ten macro-areas (Data S3). A total of 94477 SNPs were selected. Then we look for these conditions in GWAS catalog (https://www.ebi.ac.uk/gwas/) for a further description of traits and the list of the genomic variants involved in each trait (DataS3 A–B). BCFTools (v. 1.10.2) with the mpileup function was used to generate a VCF file containing genotype likelihoods in the selected positions for the previous alignment (BAM) files, setting a minimum mapping (-q) and base (-Q) quality thresholds of 20. In general, we obtained a maximum coverage of 3x for the selected SNPs, with most of them covered 1x. The obtained variants were annotated through SNPnexus (https://www.snp-nexus.org/v4/).

For the analysis of phenotypic traits related to externally visible characteristics (EVCs), 41 SNPs were obtained from the HIrisPlex-S panel^29^ and are related to eye, hair, and skin pigmentation (DataS3 A–B). The same approach described for the disease-linked traits was used to select these variants. Subsequently, the obtained variants related to the SNPs in the HIrisPlex-S panel were analyzed using the R software script provided for the HIrisPlex-S system, which allowed us to convert the results into the format required for the Hirsiplex-S online prediction model.

The variants involved in diseases genotyped for each sample are listed in DataS3 C–G. As shown in this table, several risk alleles have been detected for different diseases, but due to the low coverage and the presence of only a few risk alleles for each disease investigated, no pathological traits could be predicted with certainty. An example of this can be represented by individual 53 who, of the four risk alleles identified for Kawasaki disease^102^, has only two. Skin, eye and hair colors are the only traits for which a prediction is possible, and it was possible to attest that individual 52 had black hair and dark skin, and individuals 25, 51 and 53 had brown eyes (DataS3 D–G).

#### Strontium, Carbon, and Oxygen Analysis

Sample processing of the Pompeii tooth (Individual 25, lower premolar) took place in the Bone Chemistry Lab, Department of Anthropology, University of Florida, and all mass spectrometry was conducted in the Department of Geological Sciences, University of Florida. A small chunk of tooth enamel from Individual 25 (UF BCL 4300) was removed from the crown using an NSK dental drill and a Dedeco separating disc. The tooth enamel ‘chunk’ (~30 mg) removed was cleaned of adhering debris and dentine under a dissecting microscope, using a mounted dental drill apparatus outfitted with a carbide tungsten tapered drill bit. Two chunks of ‘cleaned’ tooth enamel (~10 mg each) were produced, one for carbon and oxygen isotope ratios using isotope ratio mass spectrometry (IRMS), and the second for strontium isotope analysis using thermal ionization mass spectrometry (TIMS).

A 10 mg sample targeted for carbon and oxygen isotope analysis was ground using an acid-cleaned agate set and tooth enamel powder was loaded into a pre-weighed 0.5 mL microcentrifuge tube. To oxidize the sample, a 2% sodium hypochlorite (NaCIO) solution was added to the sample for ~8 hours, rinsed to neutral with Milli-Q water, followed by ~8 hours of pretreatment using 0.2M acetic acid (CH_3_COOH). The pretreated sample was then rinsed to neutral with Milli-Q and the sample was placed in a −20° C freezer. Once frozen, the sample was freeze-dried for ~48 hours, and its final weight recorded prior to sample loading for IRMS. Carbon and oxygen stable isotope ratios were measured in duplicate on Sept. 6, 2017 via IRMS (Finnigan MAT 252) using a Kiel III carbonate prep device. The precision of NBS-19 standards (n=10) during the run for δ^13^C was 0.02‰ and for δ^18^O was 0.07‰.

Another 10 mg sample was targeted for radiogenic strontium ratios was transferred to a class 1000 Clean Lab in the Department of Geological Sciences, University of Florida. The tooth enamel chunk was dissolved in 50% nitric acid (HNO_3_) on a hot plate (100° C) for 24 hours in a pre-cleaned and capped Teflon^™^ vial. Vials were opened and evaporated to dryness, prior to ion chromatography. The dried residue was dissolved in 3.5 N HNO_3_ and loaded onto a cation exchange column packed with strontium-spec resin (Eichrom Technologies, Inc.) to separate the strontium from other ions. The Sr sample was then loaded onto a degassed tungsten filament and ^87^Sr/^86^Sr was measured on a Micromass Sector 54 TIMS. The sample was run for 200+ ratios at 1.5 V and normalized to ^86^Sr/^88^Sr = 0.1194 following methods outlined in^103^, against repeated analyses of the standard reference NBS-987. Obtained isotope measurements were compared against values in the literature^38–42,44^.

## Supplementary Material

DocumentS1 Document S1. Figures S1–S4 and Tables S1–S3

Dataset S1 DataS1. Supporting data for sampling, sample processing and data description, related to STAR Methods. A) Overview of sample metainformation, processing and library statistics. B) Results of AMS radiocarbon dating. C) Y-chromosomal haplogroup assignments. D) Results of relatedness analyses using BREADR and KIN.

Dataset S2 DataS2. Supporting data for population genetic analysis, related to STAR Methods. A) Individuals included in qpAdm analysis and their population labels. B) Results of qpAdm modeling using distal source populations, related to Figure 3. C) Results of qpAdm modeling using proximal source populations, shown are working models with p>=0.01. D) Results of qpAdm modeling of Individual 52 using proximal source populations.

Dataset S3 DataS3. Supporting data for phenotypic and disease risk assessment, related to STAR Methods. A) Phenotype probabilities assessed with the HIrisPlex-S system. B) List of markers used for assessment of phenotypic traits and disease risks. C) Genotyping results for disease risk markers, individual 22. D) Genotyping results for disease risk markers, individual 25. E) Genotyping results for disease risk markers, individual 51. F) Genotyping results for disease risk markers, individual 52. G) Genotyping results for disease risk markers, individual 53.

## Figures and Tables

**Table 1.** Summary of genetic results.

Cast Number	Genetic ID	Discovery Place	SNPs of 1240K set covered	Average coverage on 1240K SNP set	Genetic sex	Quantifiler TRIO Y target	Y Haplogroup	mtDNA Haplogroup	C-to-T at 5’-end (non-UDG library)	C-to-T at 5’-end (UDG library)	ANGSD X-chromosomal contamination estimate: Number of SNPs	ANGSD X-chromosomal contamination estimate: Mean	ANGSD X-chromosomal contamination estimate: C.I.
22	I3690	House of the Cryptoporticus	93,727	0.09	M	M	J2b2a1	N1b1a1	0.3	0.09	19	n/a	n/a
25	I3682	Villa of Mysteries	53,739	0.047	M	n/a*	E1b1b1b1b	H	n/a	0.1	0	n/a	n/a
50	I3683	House of the Golden Bracelet	n/a	n/a	n/a	M	n/a	H1h1	0.23	n/a	n/a	n/a	n/a
51	I3686	House of the Golden Bracelet	62,030	0.054	M	M	J2a1a4b	T2c1c	0.15	0.04	4	n/a	n/a
52	I3685	House of the Golden Bracelet	364,533	0.437	M	M	T1a1a1b2b2b1a	U1a1	0.2	0.02	350	0.0187	0–0.039
53	I3691	House of the Golden Bracelet	286,023	0.309	M	M	E1b1b	H	0.24	0.04	208	0.0086	0–0.026

* quantification not performed

**Key resources table:** 

REAGENT or RESOURCE	SOURCE	IDENTIFIER
**Biological samples**		
Ancient skeletal element	This study	I3678; Cast Number 15
Ancient skeletal element	This study	I3679; Cast Number 1
Ancient skeletal element	This study	I3680; Cast Number 79
Ancient skeletal element	This study	I3681; Cast Number 20
Ancient skeletal element	This study	I3682; Cast Number 25
Ancient skeletal element	This study	I3683; Cast Number 50
Ancient skeletal element	This study	I3684; Cast Number 21
Ancient skeletal element	This study	I3685; Cast Number 52
Ancient skeletal element	This study	I3686; Cast Number 51
Ancient skeletal element	This study	I3687; Cast Number 14
Ancient skeletal element	This study	I3688; Cast Number 54
Ancient skeletal element	This study	I3689; Cast Number 62
Ancient skeletal element	This study	I3690; Cast Number 22
Ancient skeletal element	This study	I3691; Cast Number 53
**Chemicals, peptides, and recombinant proteins**		
Distilled Water DNA free, UltraPure	Thermo Fisher Scientific	Cat# 10977035
0.5 M EDTA pH 8.0	Thermo Fisher Scientific	Cat# AM9261
Proteinase K	Thermo Fisher Scientific	Cat# AM2548
Isopropanol	Sigma Aldrich	Cat# I9516
Guanidine hydrochloride	Sigma Aldrich	Cat# G4505
Sodium Acetate Solution (3 M), pH 5.2	Thermo Fisher Scientific	Cat# R1181
Tween 20	Sigma Aldrich	Cat# P2287
Buffer PE	Qiagen	Cat# 19065
Buffer PB	Qiagen	Cat# 19066
Tris-EDTA buffer solution	Sigma Aldrich	Cat# 93283
UltraPure^™^ 0.5M EDTA, pH 8.0	Thermo Fisher Scientific	Cat# 15575020
NEB Buffer #2 10x	New England Biolabs	Cat# B7002
ATP 10 mM	New England Biolabs	Cat# P0756
BSA 20 mg/mL	New England Biolabs	Cat# B9000
dNTP Mix	Euroclone	Cat# EMR415001
USER enzyme	New England Biolabs	Cat# M5505
Uracil Glycosylase inhibitor (UGI)	New England Biolabs	Cat# M0281
T4 Polynucleotide Kinase	New England Biolabs	Cat# M0201
T4 DNA Polymerase	New England Biolabs	Cat# M0203
Bst DNA Polymerase	New England Biolabs	Cat# M0537L
Quick Ligase	New England Biolabs	Cat# M2200
PfuTurbo Cx Hotstart DNA Polymerase	Agilent Technologies	Cat# 600414
Ethanol	Merck	Cat# 1009831000
Agilent D1000 ScreenTapes	Agilent Technologies	Cat# 5067-5582
Agilent D1000 Reagents	Agilent Technologies	Cat# 5067-5583
Agilent HS Screen Tapes	Agilent Technologies	Cat# 5067-5584
Agilent HS Reagents	Agilent Technologies	Cat# 5067-5585
Agarose	Lonza	Cat# 50004
Expand Long Range dNTPack	Roche	Cat# 4829034001
Quick Blunting^™^ Kit	New England BioLaba	Cat# E1201
AccuPrime Pfx DNA Polymerase	Thermo Fisher Scientific	Cat# 12344024
Herculase II Fusion DNA Polymerase	Agilent Technologies	Cat# 600679
Sodium hydroxide Pellets	Fisher Scientific	Cat# 10306200
Dynabeads M-270 Streptavidin	Thermo Fisher Scientific	Cat# 65305
AmpliTaq Gold^™^ DNA Polymerase with Gold Buffer and MgCl2	Thermo Fisher Scientific	Cat# 4311806
Agilent aCGH Hybridization Kit	Agilent	Cat# 5188-5220
5M NaCl	Sigma Aldrich	Cat# S5150
1M NaOH	Sigma Aldrich	Cat# 71463
1 M Tris-HCl pH 8.0	Sigma Aldrich	Cat# AM9856
Quantifiler^™^ Trio DNA Quantification Kit	Thermo Fisher Scientific	Cat# 4482910
Cot-1 DNA	Invitrogen	Cat# 15279011
Dynabeads MyOne Streptavidin T1	Thermo Fisher Scientific	Cat# 65602
Salmon sperm DNA	Thermo Fisher Scientific	Cat# 15632-011
Denhardt’s solution	Sigma-Aldrich	Cat# D9905-5Ml
20x SCC Buffer	Thermo Fisher Scientific	Cat# AM9770
2x HI-RPM hybridization buffer	Agilent	Cat# 5118-5380
**Critical commercial assays**		
MinElute PCR Purification Kit	QIAGEN	Cat# 28006
QIAquick PCR Purification Kit	QIAGEN	Cat# 28104
Qubit dsDNA HS Assay Kit, 500 assays	Thermo Fisher Scientific	Cat# Q32854
High Pure Extender Assembly from the Roche High Pure Viral Nucleic Acid Large Volume Kit,40 reactions	Roche	Cat# 5114403001
QIAquick Nucleotide Removal Kit	Quiagen	Cat# 28304
MiSeq Reagent Kit v3 (150 cycle)	Illumina	Cat# MS-102-3001
NextSeq 500/550 High Output Kit v2.5	Illumina	Cat# 20024906
**Deposited data**		
Sequencing data from 5 newly reported ancient individuals	This study	ENA: PRJEB74999
Genotype data from 5 ancient newly reported individuals	This study	https://reich.hms.harvard.edu/datasets
**Oligonucleotides**		
IS1_adapter.P5: A*C*A*C*TCTTTCCCTACACGACGCTCTTCCG*A*T*C*T	Meyer & Kircher, 2010. *Cold Spring Harb Protoc* 6: pdb.prot5448^50^	Merck
IS2_adapter.P7: G*T*G*A*CTGGAGTTCAGACGTGTGCTCTTCCG*A*T*C*T	Meyer & Kircher, 2010. *Cold Spring Harb Protoc* 6: pdb.prot5448^50^	Merck
IS3_adapter.P5+P7: A*G*A*T*CGGAA*G*A*G*C	Meyer & Kircher, 2010. *Cold Spring Harb Protoc* 6: pdb.prot5448^50^	Merck
IS6: CAAGCAGAAGACGGCATACGA	Meyer & Kircher, 2010. *Cold Spring Harb Protoc* 6: pdb.prot5448^50^	Merck
IS5: AATGATACGGCGACCACCGA	Meyer & Kircher, 2010. *Cold Spring Harb Protoc* 6: pdb.prot5448^50^	Merck
Sol_iPCR-MPI: CAAGCAGAAGACGGCATACGAGAT********GTGACTGGAGTTCAGACGTGT	Kircher et al., 2012. *Nucleic Acid Research*, 40(1): e3^104^	Merck
P5_iPCR-LP: AATGATACGGCGACCACCGAGATCTACAC********ACACTCTTTCCCTACACGACGCTCTT	Kircher et al., 2012. *Nucleic Acid Research*, 40(1): e3^104^	Merck
Bio-T: Biotin-TCAAGGACATCC*G	Maricic et al., 2010. *Plos One* 5(11): e14004^22^	Merck
B: CGGATGTCCTT*G	Maricic et al., 2010. *Plos One* 5(11): e14004^22^	Merck
BO1.P5.F: AATGATACGGCGACCACCGAGATCTACACTCTTTCCCTACACGACGCTCTTCCGATCT-phosphate	Maricic et al., 2010. *Plos One* 5(11): e14004^22^	Merck
BO2.P5.R: AGATCGGAAGAGCGTCGTGTAGGGAAAGAGTGTAGATCTCGGTGGTCGCCGTATCATT-phosphate	Maricic et al., 2010. *Plos One* 5(11): e14004^22^	Merck
BO3.P7.part1.F: AGATCGGAAGAGCACACGTCTGAACTCCAGTCAC-phosphate	Maricic et al., 2010. *Plos One* 5(11): e14004^22^	Merck
BO4.P7.part1.R: GTGACTGGAGTTCAGACGTGTGCTCTTCCGATCT-phosphate	Maricic et al., 2010. *Plos One* 5(11): e14004^22^	Merck
BO5.P7.part2.F: ATCTCGTATGCCGTCTTCTGCTTG-phosphate	Maricic et al., 2010. *Plos One* 5(11): e14004^22^	Merck
BO6.P7.part2.R: CAAGCAGAAGACGGCATACGAGAT-phosphate	Maricic et al., 2010. *Plos One* 5(11): e14004^22^	Merck
BO8.P5.part1.R:GTGTAGATCTCGGTGGTC GCCGTATCATT-Phosphate	Fu et al., 2013. *Proc. Natl. Acad. Sci. USA*, 110(6): 2223–2227^56^; Fu et al., 2015. *Nature* 524:216–219^105^	Sigma-Aldrich
BO10.P5.part2.R:AGATCGGAAGAGCGTCGTGTAGGGAAAGAGTGT-phosphate	Fu et al., 2013. *Proc. Natl. Acad. Sci. USA*, 110(6): 2223–2227^56^; Fu et al., 2015. *Nature* 524:216–219^105^	Sigma-Aldrich
Sol_bridge_P5: AATGATACGGCGACCACCGA	Maricic et al., 2010. *Plos One* 5(11): e14004^22^	Merck
Sol_bridge_P7: CAAGCAGAAGACGGCATACGA	Maricic et al., 2010. *Plos One* 5(11): e14004^22^	Merck
LongR_mt1_For: GGCTTTCTCAACTTTTAAAGGATA	Meyer et al., 2007. Nucleic Acids Research 35(15)^106^	Merck
LongR_mt1_Rev: TGTCCTGATCCAACATCGAG	Meyer et al., 2007. Nucleic Acids Research 35(15)^106^	Merck
LongR_mt2_For: CCGTGCAAAGGTAGCATAATC	Meyer et al., 2007. Nucleic Acids Research 35(15)^106^	Merck
LongR_mt2_Rev: TTACTTTTATTTGGAGTTGCACCA	Meyer et al., 2007. Nucleic Acids Research 35(15)^106^	Merck
Probe for 1240K Panel	Haak et al., 2015. *Nature* 522(7555):207–11^23^; Mathieson et al., 2015. *Nature* 528(7583):499–503^55^	N/A
**Software and algorithms**		
EAGER version 1.92.55	Peltzer et al., 2016. *Genome Biol* 17:60^52^	https://github.com/apeltzer/EAGER-GUI
CircularMapper version 1.0	Peltzer et al., 2016. *Genome Biol* 17:60^52^	https://github.com/apeltzer/CircularMapper/releases
Dedup v0.12.07	Peltzer et al., 2016. *Genome Biol* 17:60^52^	https://github.com/apeltzer/DeDup
bwa v. 0.7.17-r1188	Li and Durbin, 2009. *Bioinformatics* 25(14):1754–60^58^	https://github.com/lh3/bwa
mapDamage2.0	Jónsson et al., 2013. *Bioinformatics* 29(13):1682–4^59^	https://ginolhac.github.io/mapDamage/
contamMix	Fu et al., 2013. *Curr Bio*l 23(7):553–559^60^	
Haplogrep3	Schönherr et al. 2023. *Nucleic Acids Res* 51^61^	https://haplogrep.i-med.ac.at
HID Real-Time PCR Analysis Software v1.2	Thermo Fisher	A24664
SeqPrep 1.1	https://github.com/jstjohn/SeqPrep	N/A
Preseq	Daley and Smith, 2013. *Nat Methods* 10(4):325–7^107^	N/A
ANGSD	Korneliussen et al., 2014. *BMC Bioinformatics* 15:356^64^	N/A
SAMTools	Li and Durbin, 2009. *Bioinformatics* 25(14):1754–60^58^	N/A
EIGENSOFT (version 7.2.1).	https://github.com/DReichLab/EIG	N/A
ADMIXTOOLS v.6.0	https://github.com/dReichLab/AdmixTools	N/A
KIN	Popli et al., 2023. *Genome Biol* 24(1):10^100^	N/A
BREADR	Rohrlach et al., 2023. *bioRxiv* 2023.04.17.537144^101^	N/A
sexDetERRmine	Lamnidis et al., 2018. *Nat. Commun.* 9(1):5018^62^	N/A
ADMIXTURE	Alexander et al., 2009. *Genome Res* 19(9):1655–64^75^	N/A
hapROH	Ringbauer et al., 2021. *Nat. Commun.* 12(1):5425^28^	N/A
