# Supplementary material for: Ancient DNA challenges prevailing interpretations of the Pompeii plaster casts
Source: Curr Biol. Author manuscript; Available in PMC 2024 Dec 9. (PMC11627482; doi:10.1016/j.cub.2024.10.007)
Supplement: DocumentS1 — Document S1. Figures S1–S4 and Tables S1–S3 [file NIHMS2034971-supplement-DocumentS1.pdf]

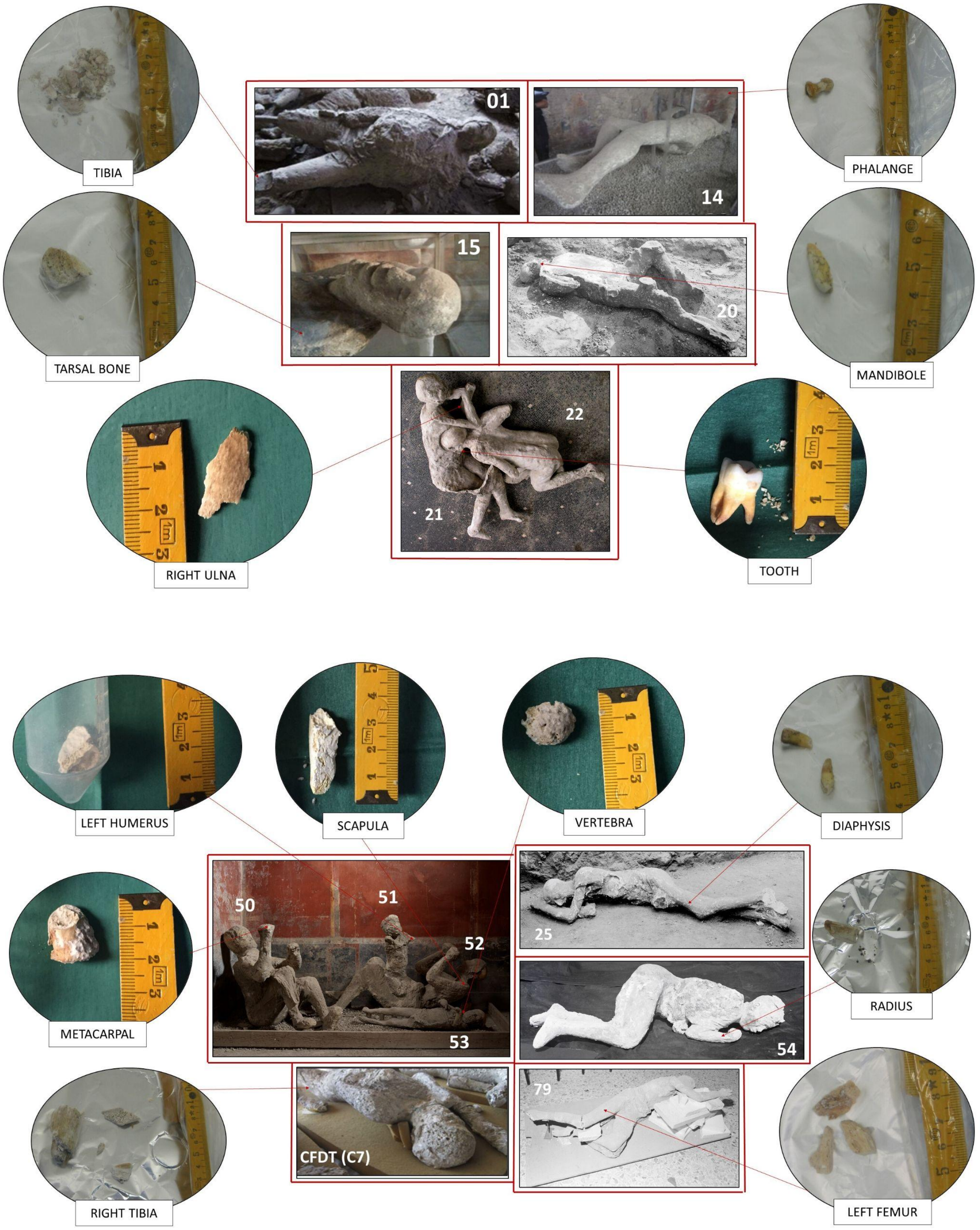

Fig. S1. Plaster casts of all the analyzed individuals and anatomical elements from which DNA was extracted, related to Figure 1 and Table 1.

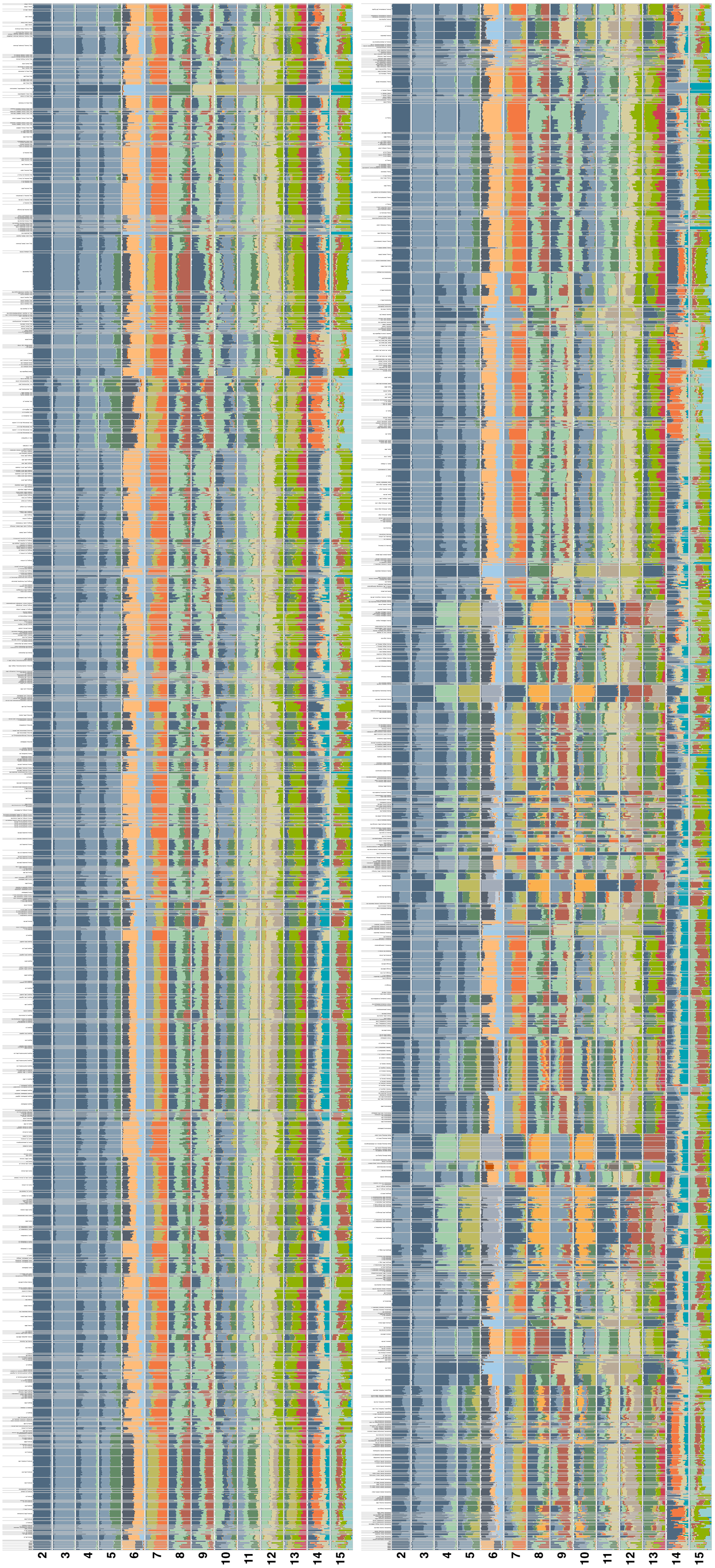

Fig. S2. Full results for ancient individuals for unsupervised ADMIXTURE analysis for k=2 to k=15, related to Figure 1. Ancestry components k shown as stacked bar plots.

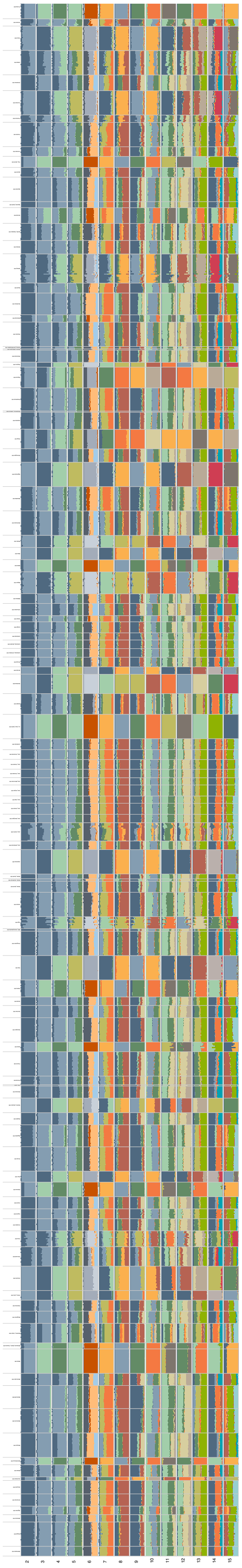

Fig. S3. Full results for modern-day individuals for unsupervised ADMIXTURE analysis for k=2 to k=15, related to Figure 1. Ancestry component k as bar plots for ancient individuals (A) and modern-day individuals (B).

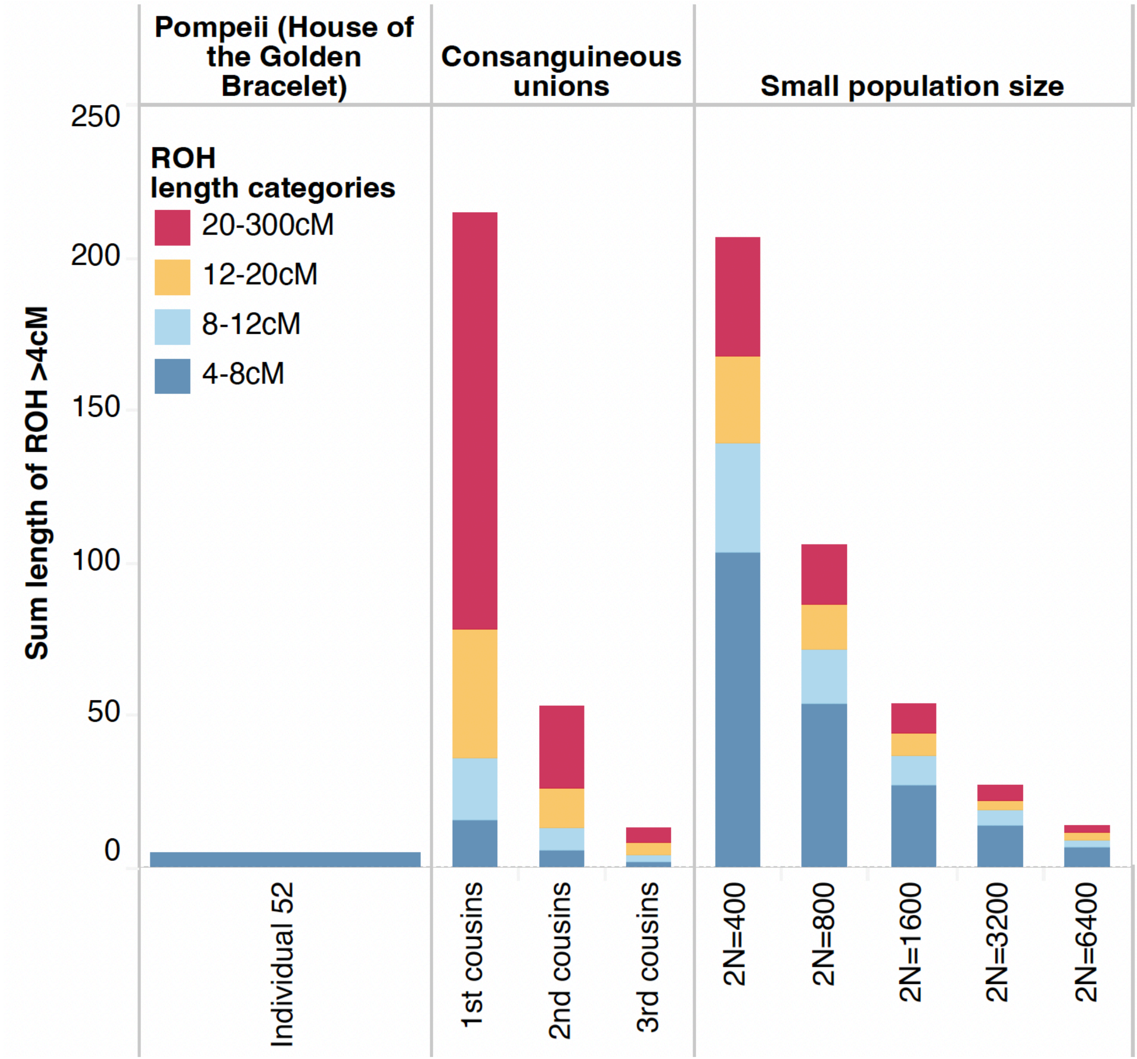

Fig. S4. ROH in individual 52, related to STAR Methods. Bar plots showing the cumulative length of ROH in four length classes (4-8, 8-12, 12-20, and >20 cM, color-coded). In individual 52 a single ROG tract of 4.71 cM was detected. The two right-most panels show the expected ROH for offspring of closely related parents and small population sizes, respectively.

**Sample ID**	**Skeletal element**	**Discovery place**
**01**	tibia	Sarno baths
**14**	phalange	Macellum
**15**	tarsal bone	Stabian baths
**20**	mandible	House of the Cryptoporticus
**21***	right ulna	House of the Cryptoporticus
**22***	tooth	House of the Cryptoporticus
**25**	diaphysis	Villa of Mysteries
**50***	metacarpal	House of the Golden Bracelet
**51***	left humerus	House of the Golden Bracelet
**52***	scapula	House of the Golden Bracelet
**53***	vertebra	House of the Golden Bracelet
**54**	radius	House of the Golden Bracelet
**CFDT (C7)**	right tibia	House of the Golden Bracelet
**79**	left femur	Sanctuary of the Blessed Virgin of the Rosary

*Samples analyzed through Quantifiler™ Trio DNA Quantification Kit (Thermo Fisher Scientific, Oyster Point, CA) and enriched for mtDNA at the University of Florence, before genome-wide analysis.

Table S1. Sample information. Sample ID, anatomical element and discovery place.

Sample ID	DNA Concentration (pg/µl)		
	LAT	SAT	YT
21	ND	ND	ND
22	ND	2.9	2.8
50	ND	28.1	22.9
51	1.2	17.8	9.3
52	6.1	117	107
53	4.3	32.7	25.4

Table S2. Trio quantification results of all samples expressed as pg/ul. LAT=large autosomal target; SAT=small autosomal target; YT= Y chromosome target. ND=not detected.

Sample	mtDNA average coverage	Percentage of position covered at least					Average fragment length	5' CtoT %	mtDNA haplogroup assignment	contammix Map Authentic
		1-fold	2-fold	3-fold	4-fold	5-fold				
**21**	0.91	57.45%	23.13%	7.58%	1.77%	0.52%	59.61	N/A	N/A	N/A
**22**	55.39	99.97 %	99.97 %	99.90 %	99.69 %	99.46%	59.46	0.3	N1b1a1	0.912
**50**	37.34	100.00 %	100.00 %	100.00 %	100.00 %	99.96%	69.26	0.23	H1h1	0.968
**51**	3.43	94.37%	81.14%	64.67%	44.61%	28.04%	69.66	0.15	T2c1c	0.977
**52**	40.82	100.00 %	100.00 %	99.92 %	99.90 %	99.87%	66	0.2	U1a1*	0.908
**53**	69.85	100.00 %	100.00 %	100.00 %	99.99 %	99.96%	58.99	0.24	H	0.987

Table S3. Results from the mtDNA capture. Results agree with the results of the independent capture performed on UDG-treated libraries in Boston (Dataset S1).
